# Fast 50 Hz Updated Static Infrared Positioning System Based on Triangulation Method

**DOI:** 10.3390/s24051389

**Published:** 2024-02-21

**Authors:** Maciej Ciężkowski, Rafał Kociszewski

**Affiliations:** Automatic Control and Robotics Department, Faculty of Electrical Engineering, Bialystok University of Technology, Wiejska St. 45D, 15-351 Bialystok, Poland; m.ciezkowski@pb.edu.pl

**Keywords:** positioning system, triangulation, autonomous navigation, IR sensors

## Abstract

One of the important issues being explored in Industry 4.0 is collaborative mobile robots. This collaboration requires precise navigation systems, especially indoor navigation systems where GNSS (Global Navigation Satellite System) cannot be used. To enable the precise localization of robots, different variations of navigation systems are being developed, mainly based on trilateration and triangulation methods. Triangulation systems are distinguished by the fact that they allow for the precise determination of an object’s orientation, which is important for mobile robots. An important feature of positioning systems is the frequency of position updates based on measurements. For most systems, it is 10–20 Hz. In our work, we propose a high-speed 50 Hz positioning system based on the triangulation method with infrared transmitters and receivers. In addition, our system is completely static, i.e., it has no moving/rotating measurement sensors, which makes it more resistant to disturbances (caused by vibrations, wear and tear of components, etc.). In this paper, we describe the principle of the system as well as its design. Finally, we present tests of the built system, which show a beacon bearing accuracy of Δφ = 0.51°, which corresponds to a positioning accuracy of ΔR = 6.55 cm, with a position update frequency of $f_{update}$ = 50 Hz.

## 1. Introduction

The use of Global Navigation Satellite Systems (GNSS) for civilian purposes has had a significant impact on the logistics industry. The ability to accurately locate people or objects has created enormous opportunities [1]. However, materials or objects in front of the receiver may scatter GNSS signals, making them unsuitable for indoor positioning. Scattering can also be an obstacle when using Bluetooth [2], Wi-Fi [3], RFID [4] and ultra-wideband (UWB) [5] technologies, which are also used for positioning. The availability of positioning solutions is expected to increase interest in this technology, which can be used for navigation, tracking, marketing, entertainment, safety, and security. Accuracy and speed are crucial in all areas of positioning. Meeting these requirements in a limited space can be challenging due to the multiple surfaces that reflect the usable signal, as well as the saturation of the space with electromagnetic waves that may interfere with the positioning system signals. Indoor and outdoor positioning differ significantly in the likelihood of encountering obstacles in the signal path. The negative effects of these obstacles can be limited by the use of other technologies. Non-line-of-sight (NLoS) refers to the obstruction of a wave’s path between the source and receiver by an object, while line-of-sight (LoS) indicates an unobstructed path [6]. Current positioning systems use information from reference points to determine the position of an object. The most common technique is distance measurement. The trilateration method requires knowledge of at least three distances, while GNSS positioning is based on measuring the difference in arrival times between two satellites, and therefore, the difference of two distances. Another method is angle measurement, which uses the triangulation method and requires knowledge of at least three angles. A technique is employed where reference points transmit a signal, either by radio or infrared, and the object’s position is determined by measuring the strength of the received signal. When talking about positioning systems, it is essential to mention those based on mobile phone technology and, in particular, on the ever-evolving 5G technology. It is expected to soon be accurate enough to allow autonomous cars to navigate with centimetre precision [7]. Recent developments and research indicate that this positioning technique, called 5G New Radio (NR), is increasingly valuable for precise indoor and outdoor positioning services. Depending on the method used, these measurements can vary from a few metres [8] to centimeters [9].

This paper describes a high-speed positioning system that uses infrared transmitters and receivers to determine position through triangulation. The paper presents the operating principle and design of the system, as well as the results of outdoor tests. The system has a measurement refresh rate of 50 Hz and achieves positioning accuracy in the centimeter range.

### 1.1. Trilateration Positioning Method

The trilateration method is a technique used to determine the location of an object based on distance measurements from at least three known points. These points are base stations with known coordinates. Assuming the distances are radii of circles, their intersection determines the common point, which is the location sought. This method is commonly used in surveying, cartography, and navigation. Although the trilateration method is simple to apply, there are various techniques available for determining positions, which are constantly evolving. A variety of wireless techniques are used for different purposes [10]. For instance, Bluetooth technology is used for locating people in office spaces [11], while Wi-Fi technology combined with received signal strength index (RSSI) analysis is used for locating pedestrians [12] or static storage tanks [13]. UWB technology, with some computational simplifications, can be used to calculate the location of robots on the production hall [14]. A two-stage trilateration algorithm using UWB works well in determining the position of drones, particularly in areas where GPS signals are not received [15]. Trilateration can also be used to locate RFID receivers [16]. Linear chirp signals are transmitted to base stations at RF frequencies to estimate the location of a tag. A trilateration system based on UAV assumptions can successfully locate and track flying insects with radio frequency tags [17]. In addition to the classical approach to the trilateration problem, many studies have proposed interesting simplifications [18] and extensions to the simultaneous and delayed algorithm [19], as well as the positioning of dynamic objects with a imprecisely known base point [20]. Other developments include an optimal trilateration algorithm based on the reptile search algorithm (RSA) for underwater wireless sensor networks [21] and K-Means clustering [22]. The algorithms’ effectiveness has been verified even in unfavorable measurement conditions, such as ambient noise, environmental interference, or uncertainty in base station coordinates.

### 1.2. Triangulation Positioning Method

The triangulation method determines the location of a point by measuring angles from known base stations located at the ends of a fixed line. The sought point is the third vertex of a triangle with one known side and two known angles. The unknown angle can be calculated using the knowledge of two known angles, as the sum of the angles of a triangle is 180 degrees. The unknown sides can be calculated algebraically.

The use of Delaunay triangulation algorithms can extend the localization method. These algorithms have been applied to localize mobile robots in forests [23] and to plan routes for mobile robots in logistics warehouses and manufacturing workshops [24]. A dynamic localization method can be one possible approach for measuring the robot’s position [25]. The mobile robot’s navigation node can be determined by measuring the RSSI [26]. Robot self-localization can be achieved by integrating trilateration and triangulation using RF sensor networks and fuzzy inference [27]. A localization model for the passive positioning of an unmanned aerial vehicle (UAV) was developed using the sine theorem, the formula for the distance between two points, and the circumferential angle theorem [28]. The triangulation method is effective in determining the distance to clouds for UAVs [29]. Triangulation is a crucial subsystem in a navigation system that reconstructs an observed 3D point using cameras with known internal parameters and positions [30]. To enhance the positioning accuracy of the triangulation method and increase its robustness against inaccuracies in received signal strength measurements, an alternative option is to use a deep neural network based on LoS classification [31]. When utilizing the triangulation method, it may be beneficial to consider using the existing infrastructure of Wi-Fi routers and mobile hotspots. A precise positioning system can be achieved by using a multi-node triangulation algorithm that takes into account the correlation between RSSI values and transmitter–receiver distance, along with a coordinate system model to determine the optimal receiver coordinates [32]. If measurements in a localization system are obtained discontinuously, such as in laser localization, an extended Kalman filter can be used. The state vector consists of angular measurements, which are dynamically estimated based on their changes. This approach eliminates approximation errors in the correction phase [33].

### 1.3. Mobile Robots Localization in Industry 4.0 Concept

Industry 4.0 and Logistics 4.0 utilize solutions that connect different production phases. Mobile robots are ideal for this purpose, as they can be integrated with an enterprise resource planning (ERP) system, enabling further automation. For instance, when the sales department places an order, the mobile robot receives a list of routes to execute sequentially. The industry employs autonomous mobile robots (AMRs), mobile manipulators (MMs), and automated guided vehicles (AGVs) for various tasks. However, the navigation methods for these vehicles differ significantly [34,35,36]. AGV systems follow predetermined routes and are guided by sensors, magnetic lines, or wires. They require complex and costly building interventions. Fault-free navigation and positioning are crucial for AMR robots to carry out tasks in various locations due to their flexibility compared to AGVs. The literature on mobile robot localization systems for the Industry 4.0 concept (see [37,38,39,40] for example, and quoted in the literature) presents standard solutions, such as trilateration and triangulation methods. To solve the navigation problem, a different approach can be taken by using visible light positioning with specially modulated LED lighting [41]. Another option is to use a system based on radio frequency identification (RFID) in the ultra-high frequency (UHF) band to determine the location of pallets carried by forklifts within a warehouse [42]. An effective localization strategy involves processing location measurements to achieve accurate recognition beyond line of sight. This can be accomplished using an enhanced particle filter based on a genetic algorithm [43]. For critical and demanding IoT device applications that require fast and reliable low-latency end-to-end (E2E) communication, the use of programmable intelligent space (PIS) is proposed as an alternative to implement atocells [44].

### 1.4. Our Previous Work, Motivations and Current Contributions

**Figure 1:**
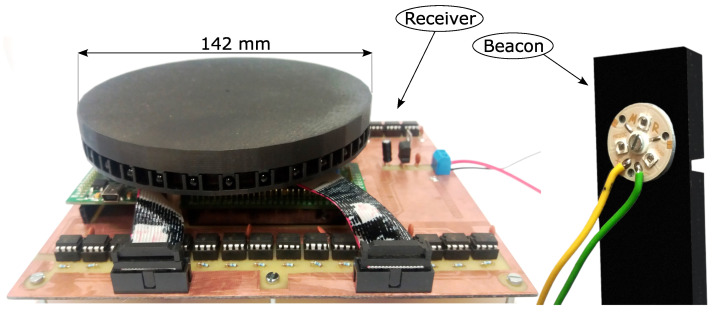
The earlier prototype of the Static Triangulation System.

Our earlier paper presented a prototype of a Static Triangulation System (STS) [45] with the following parameters: angle measurement accuracy of 0.42°, position determination accuracy of 6.97 cm, and measurement frequency of 12.5 Hz. This system is completely static, i.e., there are no moving/rotating measurement sensors, which makes it more resistant to disturbances (caused by vibrations, wear and tear on components, etc.). Another advantage of such a static system is the possibility of high position update frequencies, which in the case of systems with a rotating measurement sensor is limited by the rotational speed of the sensor. The receiver design (please look at the left part of Figure 1) was based on fabricated Discovery series prototype boards with STM32 microcontrollers. These boards were placed on the prepared PCB construction plate under the housing, which only contains the IR diodes. The overall size of this project was larger compared to the improved version presented in this paper. The operational amplifiers, using through-hole mounting technology, were placed outside the main measurement modules. This positioning could, under certain conditions, cause interference in the analogue measurement paths. The beacon in the previous prototype version consisted of three IR diodes connected in series, surface mounted on a heat sink. Three such beacons were synchronized via a wired path and required an additional microcontroller to manage the cyclic operation. Our earlier prototype of a Static Triangulation System is presented in Figure 1.

This paper presents a positioning system that improves upon the prototype. It describes a fourfold increase in position reading rate, up to 50 Hz, while maintaining angle measurement accuracy. The frequency of the position update is an important factor when considering highly dynamic and fast velocity objects. For example, a Vecna AMRs robot moves at a speed of 3 m/s, and therefore, for a positioning system operating at 10 Hz, it will move 30 cm between position updates. When the frequency of the positioning system is 50 Hz, the displacement of this robot between position readings is only 6 cm. Current laser scanning systems do not provide better measurements, despite their high cost. Commercial localization systems that use laser scanning for measurement frequency do not perform any better. For example, the ROBOSENSE system from Siman Sensors and Intelligent Machines Ltd. offers a measurement frequency of 10–40 Hz. The LaserNav Position Sensor system from Denning Branch International Robotics has a measurement frequency of 10 Hz, while the AMCO LASERNET beacon tracking system allows measurement at 20 Hz [46,47]. The updated version of the NAV2xx sensors offers a measurement frequency of up to 25 Hz [48] compared to 10 Hz in the previous version [47]. To the authors’ knowledge, other non-commercial systems using similar hardware do not achieve the same measurement frequency as the authors. The advantages of the solution in terms of compactness (external dimensions of 120 mm) and wireless synchronization of the beacons are presented in addition to the points mentioned above. The beacons have a unique design with a pentagonal shape and integrated infrared emitting diodes, providing an omnidirectional radiation source. The work area can be extended by the addition of one or more beacons. This ensures that two beacons always share each working area. Therefore, a beacon with a beam angle of 360 degrees is required.

The paper is organized as follows: Section 2 describes the basic principles of the STS positioning system, its design and the test rig used in the experimental studies; Section 3 presents the experimental results obtained under laboratory conditions (outdoor terrace); and Section 4 contains a discussion of the presented results, as well as the conclusions drawn from these results.

## 2. Materials and Methods

This chapter provides information on the methodology used in the construction of the IR transmitter–receiver triangulation system. It also describes the experimental methods used to test the positioning STS system.

### 2.1. Principle of the STS Triangulation System

**Figure 2:**
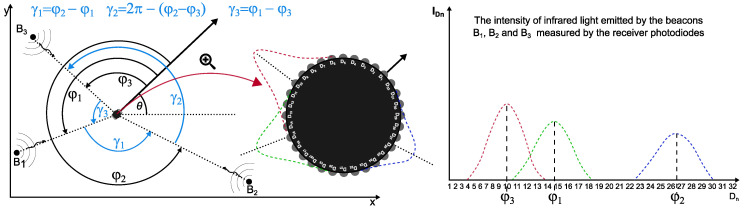
Principle of the STS triangulation system.

The principle of the STS triangulation positioning system is based on measuring the intensity of infrared light emitted by three transmitters/beacons placed at known positions $B_{i}=\left(x_{i},y_{i}\right)$. The switching of the beacons is synchronised—only one of them is active at any given time. The infrared light from the transmitter is recorded using 32 photodiodes uniformly placed around the perimeter of the circular receiver housing. The idea behind the positioning system is illustrated in Figure 2.

Two microcontrollers with 16 analogue inputs each were used to measure IR light intensity, giving a total of 32 photodiodes for measurement. The intensity of the light $I_{D_{n}}$ recorded by the *n*-th photodiode of the receiver (the black disk in Figure 2) depends on the angular orientation of the photodiode relative to the transmitting beacon $B_{i}$, resulting in the measurement curves shown on the right-hand side of Figure 2. The post-processing of the recorded measurements makes it possible to determine the angles $\varphi_{1}$, $\varphi_{2}$ and $\varphi_{3}$ which indicate the angular position of the beacons relative to the receiver. Finally, the $\varphi_{i}$ angles allow the determination of the beacon–receiver–beacon angles (denoted as $\gamma_{1}$, $\gamma_{2}$ and $\gamma_{3}$ in Figure 2) which are the input arguments to the algorithm that determines the position of the receiver in the xy plane.

### 2.2. Angle Determination Method

When the IR intensities have been recorded on the photodiodes for each of the three beacons, the angles $\varphi_{i}$ should be determined (see Figure 2). It seems natural to assume that the angle $\varphi_{i}$ is the angle for which the measured spectrum of a given beacon reaches its maximum. Unfortunately, it cannot be assumed that this angle is equal to the angular position $D_{j}$ of the *j*-th measuring photodiode for which the IR intensity is maximum because the measuring system consists of 32 photodiodes, which gives an intensity measurement every 11.25 degrees. In practice, the angle of the maximum value of the beacon spectrum will never be equal to $D_{j}$ but will be somewhere between $D_{j}$ and $D_{j+1}$. It can therefore be seen that an analysis of the measured spectrum must be carried out in order to estimate the position of the maximum intensity measured by the receiver. In our research, we tested two approaches, the results of which will be presented in this chapter.

**Figure 3:**
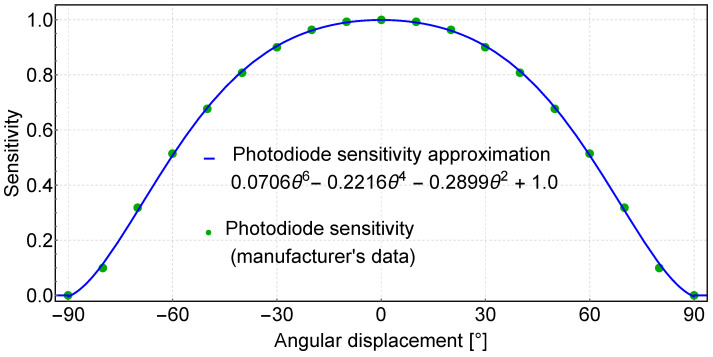
Receiver photodiode sensitivity vs. Angular displacement.

In our triangulation system, we used BPV22NF receiver photodiodes [49], for which the normalized radiant sensitivity profile plot can be found in the manufacturer’s documentation. It turns out that this profile can be described by a sixth-order polynomial:

(1) $$I_{s}\left(\theta \right)=0.0706\theta^{6}-0.2216\theta^{4}-0.2899\theta^{2}+1$$

as shown in Figure 3.

The obtained polynomial allows us to simulate the measurements recorded by the receiver photodiodes and then analyze the selected methods for the angle determination.

#### 2.2.1. Polynomial Approximation Method

Since the sensitivity profile of the receiving diode is well described by a 6th-order polynomial, it can be assumed that the approximating function will also be a polynomial of this order. Thus, the polynomial approximation method consists of finding a 6th-degree polynomial that approximates the intensity spectrum curve for the selected beacon $B_{i}$. This method requires us to first remove some data from the measured spectrum (about half of the 32 photodiodes will register zero IR intensity) and leave only those corresponding to the approximating function. Numerical approximations, such as the least squares method, can be performed for the selected data. The polynomial describing the IR intensity takes the form:

(2) $$I_{1}\left(\theta \right)=a_{0}+a_{1}\theta +a_{2}\theta^{2}+a_{3}\theta^{3}+a_{4}\theta^{4}+a_{5}\theta^{5}+a_{6}\theta^{6}$$

The purpose of the polynomial approximation method is to find such a coefficient $a_{i}$ that minimizes the cost function:

(3) $$\begin{array}{c} \underset{(a_{0},a_{1},a_{2},a_{3},a_{4},a_{5},a_{6})\in R}{\arg\min}\;\;\sum_{j=1}^{n}{\left(I_{D_{j}}-I_{1}\left(D_{j}\right)\right)}^{2} \end{array}$$

where $I_{D_{j}}$ is the intensity of an infrared light recorded by the *j*-th receiver photodiode, and $I_{1}\left(D_{j}\right)$ denotes the polynomial (2) value for $x=D_{j}$. Finally, the maximum of the approximated function $I_{1}\left(\theta \right)$ will correspond to the relative beacon–receiver angle $\varphi_{i}$.

If the sensitivity profile $I_{s}\left(\theta \right)$ of the receiving photodiodes is known, it seems reasonable to assume that the recorded beacon spectrum will simply be scaled and shifted relative to the $I_{s}\left(\theta \right)$ curve. Therefore, a different form of approximating function can be adopted, based on the known curve described by Equation (1):

(4) $$I_{2}\left(\theta \right)=AI_{s}\left(\theta -\theta_{0}\right)=A\left(0.0706{\left(\theta -\theta_{0}\right)}^{6}-0.2216{\left(\theta -\theta_{0}\right)}^{4}-0.2899{\left(\theta -\theta_{0}\right)}^{2}+1\right)$$

This function depends only on two unknown coefficients, *A* and $\theta_{0}$, of which $\theta_{0}$ is directly the sought beacon–receiver angle $\varphi_{i}$ (so there is no longer a need to search for the maximum of the function, as in the case of $I_{1}\left(\theta \right)$). In order to verify the Approximation method, both $I_{1}\left(\theta \right)$ and $I_{2}\left(\theta \right)$ approximation functions were used, as will be shown further in the paper.

#### 2.2.2. Weighted Mean of Angles Method

In the case of circular quantities, the so-called Weighted Mean of Angles method to determination of the $\varphi_{i}$ angle can be used [50]. In the case under consideration, the intensity of the measured IR radiation can be represented as a two-dimensional vector in Cartesian space $\vec{I_{D_{j}}}=\left(I_{D_{j}}\cos D_{j},I_{D_{j}}\sin D_{j}\right)$, where $I_{D_{j}}$ is the length of this vector (this is what the diode $D_{j}$ measures), and the angular position of the measuring photodiode of the receiver $D_{j}$ is the angle of inclination of the vector from the x-axis. In order to find the weighted mean value of the angle (i.e., the $\varphi_{i}$ angle), it is necessary to determine the sum of all 32 vectors $\vec{I_{D_{j}}}$, and then to determine what angle this resultant vector forms with the x-axis. The value of the angle $\varphi_{i}$ is therefore described by Equation (5).

(5) $$\varphi_{i}=arctan\frac{\sum_{j=1}^{32}I_{D_{j}}\sin D_{j}}{\sum_{j=1}^{32}I_{D_{j}}\cos D_{j}}$$

Unlike approximation, this method does not require a pre-modification of the recorded spectrum $I_{D_{j}}$ because the zero IR intensity vector contributes nothing to the vector sum. This method is much less computationally complex than the approximation method and much easier to implement on a microcontroller.

#### 2.2.3. Approximation Method vs. Weighted Mean of Angles Method

**Figure 4:**
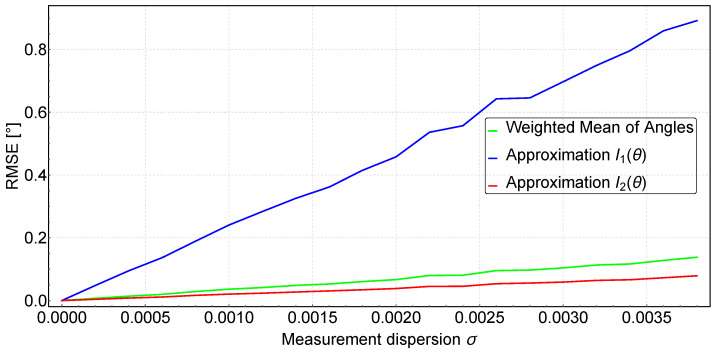
Weighted Mean of Angles vs Approximation method comparison.

The presented methods for determining the angle $\varphi_{i}$ give the same results for ideal conditions when the IR intensity measurements are undisturbed. In real-world conditions, however, measurements are always distorted by noise, which can have many sources. Typical sources of this noise are disturbances in the power supply voltage (which affects the reference voltage), temperature changes in the measurement system, electromagnetic induction (EMI) noise or the analogue-to-digital conversion process itself. To test the effect of measurement noise on the results, a simulation experiment was carried out. This consisted of generating an ideal measurement spectrum $I_{D_{j}}$ according to the mathematical model of the measurement photodiode shown in Figure 3, and then introducing noise into these measurements. The generated measurement noise was a random signal with a normal distribution and a preset standard deviation σ. Twenty values of standard deviation ranging from 0 to 0.004 with a step of 0.0002 were examined. For each value of σ, 1000 spectrum measurements were generated, for which the angle $\varphi_{i}$ was determined using both the Approximation method and the Weighted Mean of Angles method. Finally, the root mean square error (RMSE) of $\varphi_{i}$ from the 1000 spectrums for each σ was determined. The results of the simulation experiment are shown in Figure 4.

As can be seen in Figure 4, the RMSE error increases linearly with increasing dispersion of the measurements, but for the Approximation method with the fitting function $I_{1}\left(\theta \right)$ described by Equation (2), it increases about five times or even more than in the other two cases. The Approximation method with fitting function $I_{2}\left(\theta \right)$ gives the best results, while the results for the Weighted Mean of Angles method are slightly worse than Approximation $I_{2}\left(\theta \right)$ (less than 0.1 degrees).

If the analog measurement is performed by an STM32 family microcontroller with a resolution of 12 bits, the dispersion of the measurement ranges from 10 to 20 LSB (for normalized measurement: $LSB=1/2^{12}=0.00024$), which gives a σ in the range of 0.0024–0.0048 [51]. As can be seen in Figure 4, for such a σ dispersion range, the value of the $\varphi_{i}$ angle determined by the Approximation $I_{2}\left(\theta \right)$ method or the Weighted Mean of Angles method is much more accurate than using the Approximation method with the fitting function $I_{1}\left(\theta \right)$.

Due to the fact that the post-processing calculations take place directly on the receiver’s on-board microcontroller, it was decided to choose the Weighted Mean of Angles method, which, despite its simplicity, gives acceptable uncertainties, not much higher than the much more computationally complex (and requiring prior data preparation, i.e., removal and rearrangement) Approximation $I_{2}\left(\theta \right)$ method.

### 2.3. IR Beacon Transmission System

The infrared beacon consists of an array of five PK2S-3LJE-A high-power diodes placed on the sides of a pentagon-shaped aluminium heat sink. The beacon diodes emit infrared with a wavelength of 850 nm and are characterized by a Lambert radiation pattern, a viewing angle of $140^{\circ }$ and a radiometric power for 1 A current of approximately 1560 mW [52]. Based on the manufacturer’s data, the normalized intensity of a single beacon diode can be described by the approximated equation:

(6) $$I_{B}\left(\theta \right)=1-0.0484\theta^{2}-0.0467\theta^{4}-0.1931\theta^{6}+0.0625\theta^{8}\;\mathrm{for}\;-\pi /2<\theta <\pi /2$$

**Figure 5:**
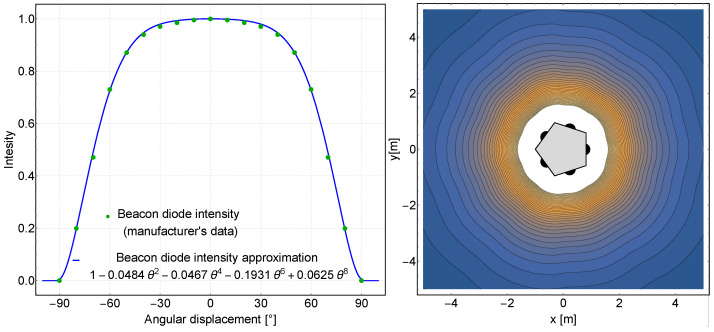
Beacon radiation pattern.

The fit of the manufacturer’s data to the polynomial is shown on the left-hand side of Figure 5.

The approximate normalized irradiance distribution of a single transmitting diode can therefore be written using the inverse square law:

(7) $$E_{B}\left(\theta ,r\right)=\frac{I_{B}\left(\theta \right)}{r^{2}}$$

where *r* is the distance from the transmitting diode. For five IR transmitting diodes placed on the sides of the pentagon, the total irradiance distribution for the transmitting beacon is obtained as on the right-hand side of Figure 5.

**Figure 6:**
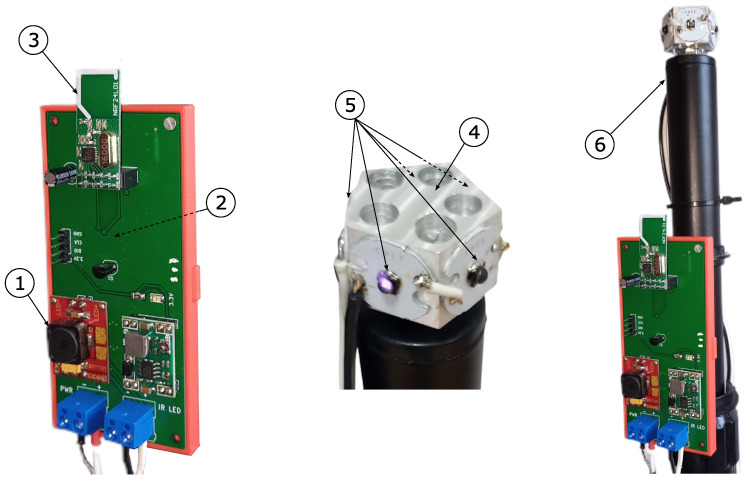
Hardware configuration of the IR beacon transmission system.

To avoid interference with each other’s transmitted signals, the beacons operate in a synchronized cycle, so that only one beacon is operating at a time. To ensure this operation, the beacon system is equipped with a microcontroller control system, which is shown on the left side of Figure 6. The control device consists of a current driver, providing a constant current of 900 mA flowing through the transmitting diodes (see ➀ in Figure 6), which is managed by the STM32G030F6 microcontroller (➁ in Figure 6 on the reverse side of the PCB) [53]. The microcontroller is also responsible for controlling the nRF24L01 radio transceiver module marked as ➂ which is used to synchronise the beacons operation [54]. The transmitting diodes (➄ in Figure 6) placed on the heatsink ➃ together with the control device, were finally placed on the mast ➅ forming the transmitting beacon, as shown on the right side of Figure 6.

Each of the three beacons $B_{i}$ in the triangulation system has its own unique number $ID_{i}$. In the synchronization cycle, the first active beacon is $B_{1}$ then $B_{2}$ and then $B_{3}$. The first beacon $B_{1}$, when it is activated, turns on the IR diodes for 5 ms and sends a radio message using nRF24L01, which contains its ID number. The rest of the beacons, meanwhile, listen to radio messages sent by the nRF24L01 modules. When beacon $B_{2}$ receives a message from beacon $B_{1}$, it waits 5 ms and then turns on the IR diodes for 5 ms and sends a message with its own ID number. Beacon $B_{3}$ responds to the message from beacon $B_{2}$ in the same way that $B_{2}$ responds to $B_{1}$. When beacon $B_{1}$ receives a signal from $B_{3}$, it waits 5 ms until beacon $B_{3}$ finishes its work, and then waits another 5 ms. During these next 5 ms, neither of the beacons is working, which allows us to record the background IR radiation from sources other than the beacons. Then, the whole cycle repeats, and its time is equal to 3 × 5 ms (beacon activity) + 5 ms (background measurement) = 20 ms. The frequency of the beacon’s synchronization cycle is therefore 50 Hz, which makes it possible to determine the actual position of the receiver at a frequency just equal to 50 Hz.

**Figure 7:**
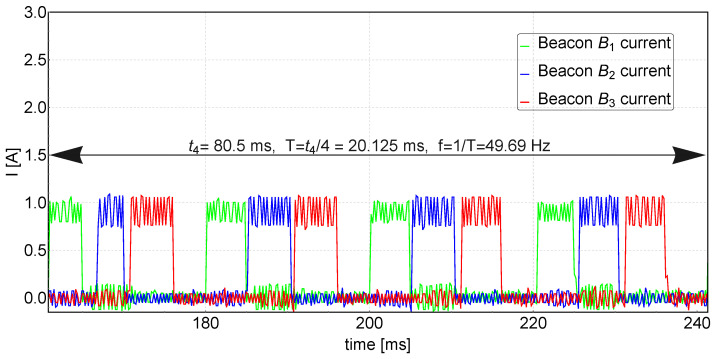
Oscilloscope diagrams of beacon IR diode currents.

Figure 7 displays the current waveforms supplied to the diodes of each beacon during their activation sequences. The average current value is approximately 900 mA, while the oscillations, with a frequency of 2.5 kHz are a result of the converter operating in a buck configuration. The stations are cycled in the assumed sequence, starting from $B_{1}$, and their synchronization frequency is close to the expected value of 50 Hz.

### 2.4. IR Receiver

**Figure 8:**
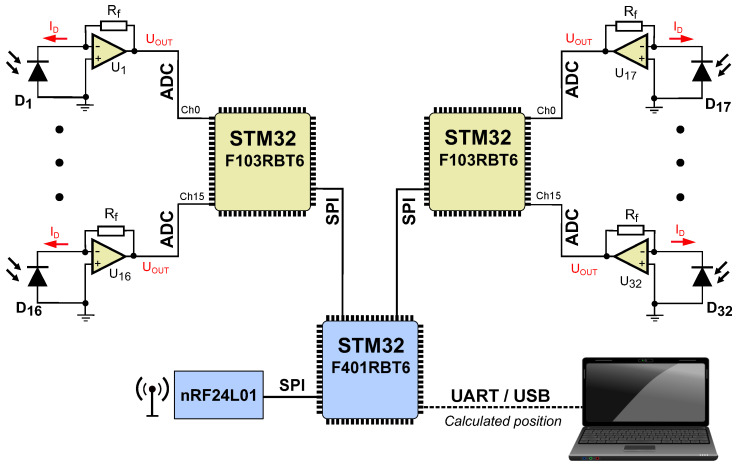
Analog front-end and digital part of the IR receiver.

**Figure 9:**
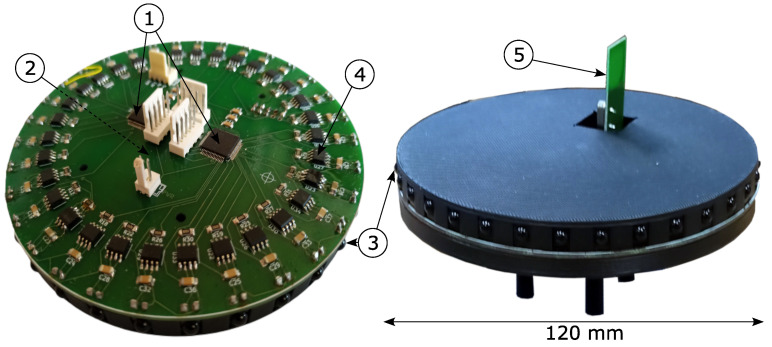
Hardware configuration of the IR receiver.

A simplified block diagram of the analog and digital parts of the 32-channel IR receiver and its implementation is shown in Figure 8 and Figure 9, respectively. The receiver was housed in a compact 3D-printed casing, which contained a two-layer PCB with electronic components. On the top side of the PCB, two microcontrollers ➀ were located, which measured the voltages in the analog channels. The STM32 microcontrollers’ internal ADCs were used. The ADC converter has a maximum clock frequency of 35 MHz, resulting in a minimum conversion time of 400 ns for 12-bit resolution. Circuit measurements are taken at a sampling rate of 1 kHz. The photodiodes ➂ are positioned at 11.25-degree intervals and operate in the photovoltaic mode with MCP6271 transimpedance amplifiers ➃ ($U_{1}$,...,$U_{32}$) in typical configuration [55]. Each amplifier converts a small current $I_{D}$ into a corresponding voltage, which is then applied to the ADC input. The advantage of the use of the photovoltaic mode is the reduction of the dark current. In typical diodes, the application of a barrier polarization voltage increases the reverse current. A similar relationship applies to the dark current in photodiodes. The greater the voltage of barrier polarization, the greater the dark current, while without polarization, it is eliminated. Furthermore, this mode enables the photodiode to be applied in pulsed circuits, making it ideal for precision applications. The circuit’s gain is determined by the feedback resistor $R_{f}=820\;k\Omega \;$ (tol.±0.1%), and the amplifier output voltage is equal to $U_{OUT}=I_{D}\cdot R_{f}$. The operational amplifier is a rail-to-rail type. This means that its maximum output voltage is equal to its supply voltage of 3.3 V. The microcontrollers ➀ communicate with ➁ via the SPI interface, with a clock signal of 1 kHz. The bus transmits measurement results from 32 channels. The microcontroller ➃ communicates with the radio module ➄ through the SPI interface, which is the wireless component of the beacon synchronizing circuit. For diagnostic purposes and in order to read out the calculated coordinates and angles, the UART interface is used by microcontroller ➁.

The IR receiver is equipped with an nRF24L01 radio transceiver module marked ➄ in Figure 9. This radio module operates in listen-only mode so that the receiver has information about the current status of the beacon’s duty cycle. When the module receives information with the $ID_{i}$ number of a particular beacon, after a period of approximately 2.5 ms (half of the beacon’s activity period) from receiving the $ID_{i}$, it registers the data from the 32 photodiodes as a spectrum of the beacon $B_{i}$. In our previous version of the system, the beacons were synchronised by wire using an additional circuit. The previous receiver had no connection or feedback to the beacon system and had to identify the beacon signal (which signal was coming from which beacon) by analyzing the recorded spectrum, which required more time, leading to a system operating frequency below 50 Hz. When spectrum from all beacons is recorded, the IR receiver determines the $\varphi_{i}$ angles via the Weighted Mean of Angles method described in Section 2.2.2.

To determine the position of the receiver, a method belonging to the family of Geometric Circle Intersection algorithms, the so-called ToTal algorithm, was used [56]. The algorithm consists of two computational phases. In the first phase, the equations of the three circles are determined based on the known positions of beacons $B_{1}$, $B_{2}$, $B_{3}$ and the measured angles $\varphi_{1}$, $\varphi_{2}$ and $\varphi_{3}$. If *R* is the position of the receiver, the given circle passes through the positions of the beacons (e.g., $B_{1}$ and $B_{2}$,) and the point *R*. Three beacons and three angles give three circles, whose centers and radii can be determined analytically. The second phase of the ToTal algorithm determines the intersection of the three circles obtained from phase one. It turns out that it is more convenient to calculate by finding the intersection of the straight lines, known in geometry as radical axes or power lines. The determined intersection of these lines is the sought position of the receiver.

### 2.5. Laboratory Experimental Set-Up

**Figure 10:**
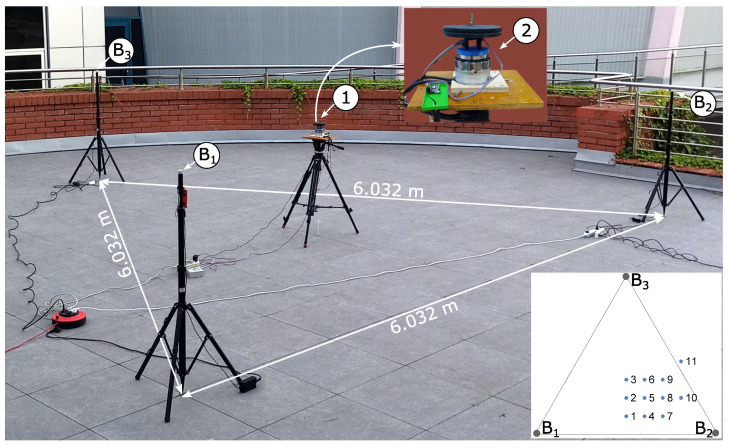
Laboratory experimental set-up.

The measurement experiment of the STS triangulation positioning system was carried out on the terrace of the Faculty of Electrical Engineering of the Bialystok University of Technology. As these were the first experimental tests on the constructed system, it was ensured that there were no obstacles in the working area (and its close vicinity) that could affect the measurement result. It is well known that in such measurement systems, reflections of signals from objects (e.g., walls) interfere with the measurement spectrum; therefore, an error occurs in the determination of the beacon–receiver angles. The beacons $B_{1}$, $B_{2}$ and $B_{3}$ were arranged in an equilateral triangle at positions $B_{1}$ = (0.0, 0.0) m, $B_{2}$ = (6.032, 0.0) m, $B_{3}$ = (3.016, 5.223) m as shown in Figure 10. The IR receiver of the positioning system ➀ was placed on a tripod. To enable measurements for different orientations of the receiver disk, it was placed on a servo motor (see ➁ in Figure 10). Measurements were therefore taken for a given tripod position and for different orientations of the receiver, which was rotated every 5 degrees. For each given IR receiver position (defined by tripod position and receiver disk orientation), 10 measurements were taken, which gives a total of 10 × 360/5 = 720 measurements for the given tripod position. The measurement experiment was performed for the eleven tripod positions marked (from 1 to 11) in Figure 10.

Based on the experimental measurement data, the following statistical parameters were calculated for each tripod position: average position in x-direction $\bar{x}$; average position in y-direction $\bar{y}$; standard deviation $\sigma_{x}$ of the position in the x-direction; standard deviation $\sigma_{y}$ of the position in the y-direction; standard deviation $\sigma_{R}$ of the position $R=\sqrt{x^{2}+y^{2}}$; and root mean square error of the position *R* define as:

(8) $${rmse}_{R}=\sqrt{\frac{\sum_{j=1}^{n}{\left(x_{real}-x_{j}\right)}^{2}+{\left(y_{real}-y_{j})\right)}^{2}}{n}}$$

where $\left(x_{real},y_{real}\right)$ is the real triod position, $\left(x_{j},y_{j}\right)$—measured triod position and *n*—number of measurements in a given tripod position (n=720); standard deviation $\sigma_{\gamma }$ of the angles $\gamma_{i}$ defined as:

(9) $$\sigma_{\gamma }=\sqrt{\frac{\sigma_{\gamma_{1}}^{2}+\sigma_{\gamma_{2}}^{2}+\sigma_{\gamma_{3}}^{2}}{3}}$$

and root mean square error of the measured angles $\varphi_{i}$:

(10) $${rmse}_{\varphi }=\frac{1}{2}\sqrt{\frac{{rmse}_{\gamma_{1}}^{2}+{rmse}_{\gamma_{2}}^{2}+{rmse}_{\gamma_{3}}^{2}}{3}}$$

The determined parameters are presented in table form in Section 3.

## 3. Results

**Figure 11:**
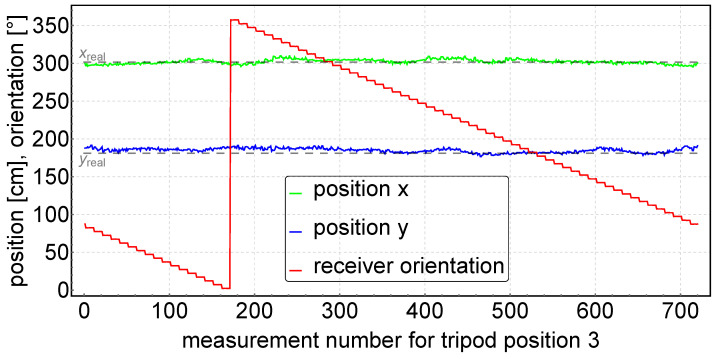
Experimental results for the tripod in position 3.

This section describes and discusses the results of the experimental studies. The measured data for measurements at tripod positions 1 to 11 (see Figure 10) were statistically processed and then presented in Table 1. Figure 11 shows an example of the experimental results: the determined positions and receiver orientations for the tripod in position 3.

Based on the experimental data shown in Table 1, the accuracy of measurement of the angle φ was 0.51°. This is comparable to the other triangulation systems presented in the papers [57] (Δφ = 0.6°, $f_{update}$ = 10 Hz), [58] (Δφ = 2.0°, $f_{update}$ = 10 Hz), [59] (Δφ = 1.5°, $f_{update}$ = 1 Hz) and [47] (Δφ = 0.24°, $f_{update}$ = 10 Hz). However, the update frequency in our STS triangulation system, which is $f_{update}$ = 50 Hz, is several times higher than in the above-mentioned systems. In addition, our STS system contains no moving parts, and is therefore simpler to build and more resistant to disturbances. The most important indicator of the triangulation system accuracy is the precision of angle determination. This accuracy translates directly into positioning accuracy, which averaged ΔR = 6.55 cm in our experiment. Position accuracy decreases with distance from the centre of the triangle, as can be seen in the $rmse_{R}$ column in Table 1. It should be emphasized that the data in Table 1 come from an experiment where there were no obstacles in the close vicinity of the system, so reflections of the IR signals were minimized. In such measurement systems, signal reflections from objects (e.g., walls) interfere with the measurement spectrum and affect positioning accuracy.

## 4. Conclusions

In this paper, we presented the concept of a static triangulation positioning system (STS) that operates at a refresh rate of $f_{update}$ = 50 Hz. We demonstrated the basic principles of the system’s operation, described its main components and carried out a preliminary theoretical simulation analysis, which confirmed the correctness of the chosen concept and the hardware components used in the designed devices. In the following sections, we described the measurement experiment carried out, and based on the recorded data, we determined the accuracy of our positioning triangulation system, which was Δφ = 0.51°, and translated to an accuracy of ΔR = 6.55 cm in position. It turned out that the system we built, despite not containing any moving/rotating sensors (as is commonly used), had comparable measurement accuracy to other positioning triangulation systems, and moreover, is a couple of times faster in terms of the refresh rate of the determined position, which was $f_{update}$ = 50 Hz.

However, the system still needs further modification. Based on the measurement data, it was noted that for measurements with a tripod in a fixed position with the receiver, the calculated position is described by a dispersion $\sigma_{R}$ of up to 5 cm. The likely reason for this non-isotropy due to the angular position of the receiver is the differences in the characteristics of the measurement photodiodes mounted on the receiver disk. In our future research, we want to investigate what accuracy and isotropy will be achieved by a receiver composed of photodiodes that do not contain factory-mounted plastic lenses, as in the BPV22NF diodes (produced by Vishay Intertechnology, Inc, Malvern, PA, USA) we used. We suspect that the lenses in the diodes may be the source of discrepancies in their characteristics. 

## Figures and Tables

**Table 1:** Experiment results.

Tripod Position	$x_{\mathrm{real}}$ [cm]	$y_{\mathrm{real}}$ [cm]	$\bar{x}$ [cm]	$\bar{y}$ [cm]	$\sigma_{x}$ [cm]	$\sigma_{y}$ [cm]	$\sigma_{R}$ [cm]	${\mathrm{rmse}}_{R}$ [cm]	$\sigma_{\gamma }$ [°]	${\mathrm{rmse}}_{\varphi }$ [°]
1	301.6	60.32	303.53	61.59	4.62	2.35	4.47	5.67	0.36	0.48
2	301.6	120.64	302.75	122.91	3.36	2.35	2.99	4.83	0.4	0.49
3	301.6	180.96	302.26	184.03	2.39	2.63	2.31	4.74	0.43	0.48
4	361.92	60.32	361.91	62.32	4.24	2.13	3.99	5.14	0.46	0.46
5	361.92	120.64	361.62	122.83	4.09	3.13	3.32	5.61	0.37	0.53
6	361.92	180.96	364.01	184.64	3.06	2.46	2.13	5.77	0.35	0.61
7	422.24	60.32	421.93	60.84	5.72	3.02	5.32	6.49	0.45	0.46
8	422.24	120.64	421.94	122.67	5.16	3.74	4.21	6.69	0.5	0.55
9	422.24	180.96	422.2	182.99	4.06	4.85	2.45	6.64	0.47	0.51
10	482.56	120.64	480.69	122.96	5.24	4.9	4.01	7.76	0.42	0.47
11	482.56	241.28	479.56	247.7	4.62	6.36	2.34	10.57	0.44	0.51
**Pooled:**					**4.34**	**3.68**	**3.56**	**6.55**	**0.42**	**0.51**

## Data Availability

Dataset available on request from the authors.
